# The Impact of Nutrition on DNA Methylation: Methodological Challenges in Understanding Cause and Effect

**DOI:** 10.3390/nu18152453

**Published:** 2026-07-27

**Authors:** Yusha Araf, Theo Portlock, Justin M. O’Sullivan

**Affiliations:** 1 Liggins Institute, The University of Auckland, Auckland 1023, New Zealand; yusha.araf@auckland.ac.nz (Y.A.); theo.portlock@auckland.ac.nz (T.P.); 2 Maurice Wilkins Centre for Molecular Biodiscovery, Auckland 1010, New Zealand; 3 MRC Lifecourse Epidemiology Unit, University of Southampton, Southampton SO16 6YD, UK

**Keywords:** DNA methylation, nutrition, epigenetics, malnutrition, causal inference, epigenetic biomarkers, long-read sequencing, multi-omics, digital twin, precision nutrition

## Abstract

DNA methylation is a key epigenetic mechanism linking nutritional exposures to gene regulation and downstream phenotypes. Both undernutrition and overnutrition are associated with distinct methylation signatures, some of which persist beyond the initial exposure window and may relate to long-term metabolic, immune, and neurodevelopmental outcomes. However, the extent to which these associations reflect causal mechanisms, adaptive responses, or secondary effects remains unresolved. Here, we synthesize current evidence on how nutrition influences DNA methylation across the life course, integrating biochemical pathways, metabolic signaling, and microbiome-derived processes within a unified framework. We highlight how these diverse inputs converge on core regulatory axes, including methyl donor availability, enzyme activity, and chromatin context. We then evaluate emerging long-read sequencing, single-cell methylomics, deconvolution strategies, and multi-omic integration methodologies that are improving cellular resolution and enabling a more mechanistic interpretation of nutritional epigenetic variation. Despite these advances, major challenges remain, including tissue specificity, measurement limitations, and the difficulty of distinguishing causation from correlation in observational data. We argue that progress will depend on longitudinal and interventional study designs, improved causal inference frameworks, and integration of functional validation with high-resolution molecular profiling. Addressing these challenges will be critical for determining whether nutrition-associated methylation changes represent biomarkers, mediators, or causal drivers of disease, and for translating epigenetic insights into precision nutrition strategies.

## 1. Introduction

Beyond the DNA sequence, epigenetic modifications are widely recognized as key regulatory mechanisms linking environmental exposures to gene expression and phenotype [1,2]. DNA methylation at cytosines (5mC) is dynamically regulated by three intersecting determinants: the enzymatic machinery that writes, maintains, and removes methyl marks; methyl-donor availability from one-carbon and methionine-cycle metabolism; and the local chromatin context that determines whether CpG sites are accessible to these enzymes [3,4,5,6,7,8]. DNA methyltransferases (DNMTs) establish and maintain 5mC, whereas ten-eleven translocation (TET) enzymes oxidize 5mC to 5hmC and further derivatives during active or replication-dependent demethylation [4,5,9,10]. The methylation reaction uses S-adenosylmethionine (SAM) as the methyl donor and generates S-adenosylhomocysteine (SAH), making the SAM:SAH balance a direct biochemical link between nutritional state and cellular methylation capacity [6,7,11]. Chromatin structure, histone modifications, nucleosome positioning, and transcription-factor occupancy further shape where methylation can be gained, maintained, or lost [1,5,8,12]. As such, variation in DNA methylation has been widely associated with disease risk and phenotypic diversity [2,5,12,13].

Diet is increasingly recognized as an important determinant of DNA methylation. Nutritional exposures are associated with methylation variation, altered gene regulation, and differences in disease risk across populations [14,15,16,17]. Emerging approaches, including Mendelian randomization, provide limited support for potential causal relationships linking genetically instrumented dietary exposures to differential methylation at loci associated with cardiometabolic outcomes [18,19]. However, the broader causal architecture linking nutrition, epigenetic regulation, and disease remains incompletely resolved.

Understanding these relationships is increasingly important in the context of modern dietary patterns, which are characterized by interacting system-level disruptions. These include chronic energy excess and metabolic overload, reduced dietary fiber intake and microbiome perturbation, widespread consumption of ultra-processed foods, micronutrient dilution, temporal mismatches between feeding and circadian biology, and the global coexistence of early-life undernutrition and food insecurity. Collectively, methyl donor availability, enzyme activity, and nutrition-responsive signaling pathways reshape metabolic, inflammatory, and microbial signaling environments that are known to influence epigenetic regulation [7,20].

Despite substantial progress in identifying nutrition-associated methylation signatures, a key challenge remains: how to move from largely associative observations toward mechanistically interpretable and clinically actionable models? While previous studies have characterized individual pathways and exposures, fewer frameworks integrate nutritional complexity, biological mechanisms, and longitudinal dynamics to define causal relationships between diet, epigenetic regulation, and disease risk. Moving to a causal relationship, where altering X leads to a change in Y, all else being equal, is critical for the study of nutrition and epigenetics. Such a movement will clarify that changing diets (nutrition) directly causes changes in the methylation pattern and long-term disease risk.

Addressing this gap will require approaches that combine mechanistic insight with longitudinal and multi-omic data to resolve the pathways through which nutritional exposures shape epigenetic states and downstream phenotypes. A clearer understanding of this causal architecture represents a critical step toward translating nutritional epigenetics into predictive and intervention-focused precision health strategies.

## 2. How Is Nutrition Associated with DNA Methylation?

**Figure 1:**
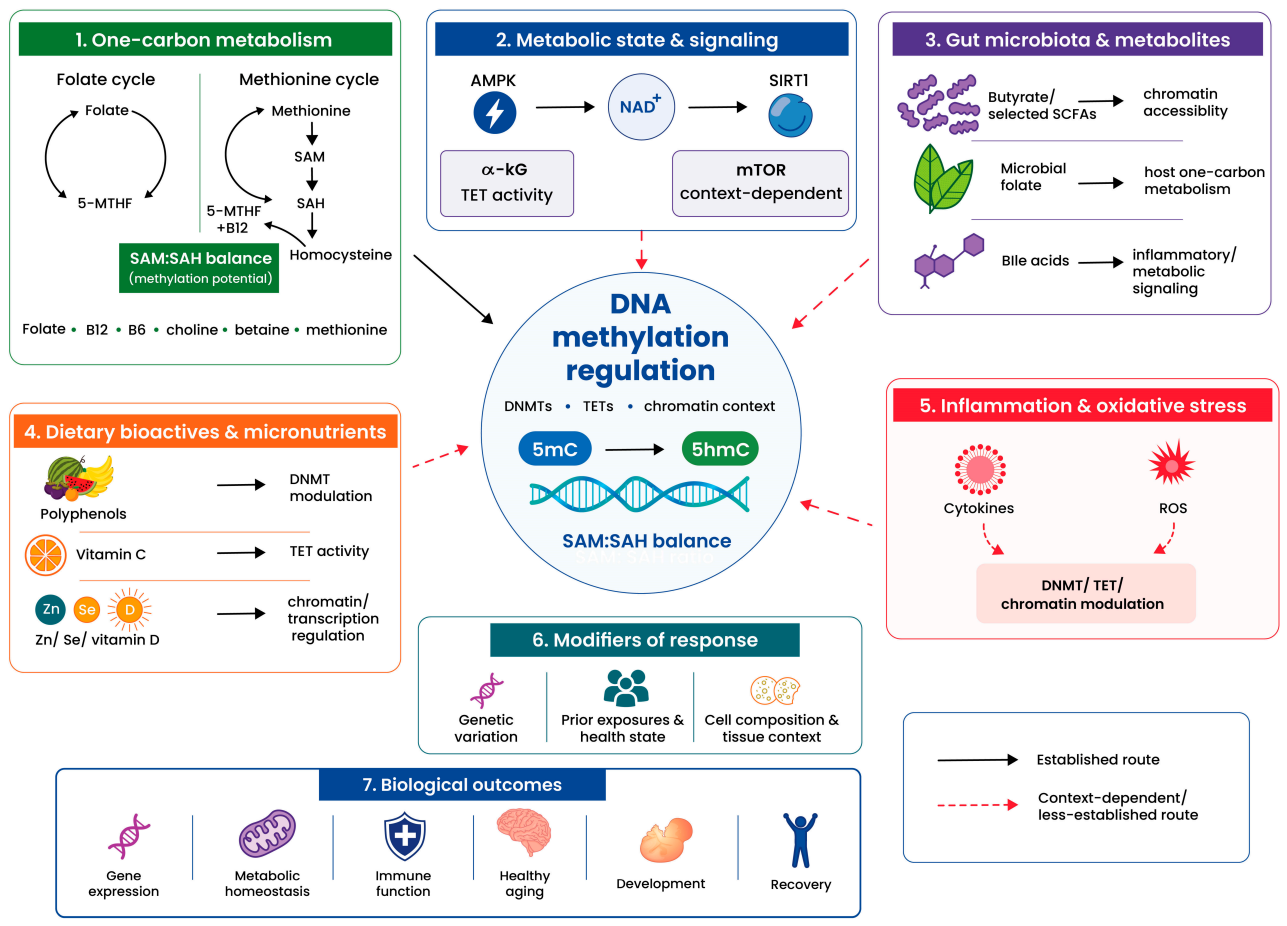
Nutritional and physiological determinants of DNA methylation regulation. Nutritional components may influence DNA methylation through several converging biochemical and physiological routes. (1) Methyl donor nutrients, including folate, vitamin B12, vitamin B6, choline, betaine and methionine, support one-carbon and methionine-cycle metabolism, thereby regulating methyl donor status and the SAM:SAH balance. (2) Energy balance and nutrient state influence metabolic signaling through AMPK, NAD^+^, SIRT1, α-ketoglutarate and mTOR, which can modify DNMT activity, TET activity and chromatin context. (3) Microbiota-derived routes, including butyrate and selected short-chain fatty acids, microbial folate and bile-acid metabolism, connect diet with chromatin accessibility, host one-carbon metabolism and inflammatory or metabolic signaling. (4) Dietary bioactives and micronutrients, including polyphenols, vitamin C, zinc, selenium and vitamin D, may modulate DNMT activity, TET activity and chromatin or transcriptional regulation. (5) Nutrition-related inflammatory and oxidative states, including cytokine signaling and reactive oxygen species, may influence DNMT/TET activity and chromatin regulation in a context-dependent manner. (6) Response modifiers include genetic variation, prior exposures, health state, cell composition and tissue context; these factors may influence biological responses or the observed methylation signal but are not represented as a single causal pathway. (7) Biological outcomes associated with DNA methylation variation include gene expression, metabolic homeostasis, immune function, healthy aging, development and recovery. These routes converge on DNA methylation regulation through DNMTs, TETs, chromatin context and the SAM:SAH ratio. Solid black arrows indicate established routes; dashed red arrows indicate that the evidence supporting these routes is weaker than seen for the solid black arrows.

Multiple interconnected pathways integrate dietary inputs into epigenetic responses (Figure 1). First, one-carbon and methionine-cycle metabolism provide the central methyl-donor pathway for DNA methylation by regulating methionine availability, S-adenosylmethionine (SAM) production, and SAM:SAH balance (Figure 1(1)) [6,14,21]. Second, nutrient and energy-sensing pathways, including AMPK, NAD^+^/SIRT1, α-ketoglutarate-dependent dioxygenase activity, and mTOR signaling, connect cellular metabolic state to DNMT activity, TET activity, and chromatin regulation (Figure 1(2)) [22]. Third, the gut microbiota can modulate methylation-related pathways through dietary fiber-derived short-chain fatty acids, microbial folate production, bile-acid metabolism, and systemic inflammatory or metabolic signaling (Figure 1(3)) [23,24]. Fourth, dietary bioactives and micronutrients, including polyphenols, vitamin C, zinc, selenium, and vitamin D, can influence DNA methylation by modulating DNMT activity, TET activity, antioxidant pathways, and chromatin accessibility (Figure 1(4)) [22]. Fifth, nutrition-related oxidative stress and inflammation can reshape methylation patterns by altering redox status, SAM availability, cytokine signaling, DNMT/TET activity, and tissue or immune-cell composition (Figure 1(5)) [22,25]. Together, these pathways converge on three major regulatory axes: SAM:SAH balance, DNMT/TET activity, and chromatin context [22]. However, it should be noted that these pathways are supported by varying levels of evidence and are not necessarily causally equivalent. Moreover, the evidence is not equivalent, as some is derived from direct biochemical relationships, in vitro studies, animal studies, observational studies, or intervention studies. In the following sections, we have distinguished established routes from context-dependent or still-emerging mechanisms [25,26].

### 2.1. One-Carbon Metabolism

One-carbon metabolism is the pathway most associated with DNA methylation and nutrition-sensitive methyl-donor supply [6,14,21]. Methionine availability provides the most direct entry point into the methylation cycle. L-methionine is converted by methionine adenosyltransferase to S-adenosylmethionine (SAM), the universal methyl donor used by DNA methyltransferases (DNMTs) and other methyltransferases [6,11]. After donating its methyl group, SAM becomes S-adenosylhomocysteine (SAH), which is hydrolyzed to homocysteine and adenosine. Homocysteine can then be remethylated to methionine through either the folate/vitamin B12-dependent methionine synthase pathway or the choline/betaine-dependent betaine-homocysteine methyltransferase pathway [6,21]. Alternatively, homocysteine can enter the vitamin B6-dependent transsulfuration pathway through cystathionine beta-synthase and cystathionine gamma-lyase, linking methylation capacity to glutathione synthesis, redox balance, and sulfur-amino-acid metabolism [6,11,27]. Thus, the combined dietary availability of methionine, folate, vitamin B12, vitamin B6, choline, and betaine determines the biochemical substrate environment that supports SAM production, SAM:SAH balance, and DNMT-mediated DNA methylation (Figure 1(1)).

This methionine–SAM–SAH–homocysteine cycle is central because SAH is a product inhibitor of methyltransferases; therefore, low SAM, high SAH, or a reduced SAM:SAH ratio can reduce methylation potential even when DNMT protein abundance is unchanged [6,7,11]. Primary studies support this mechanism at several levels. Maternal methyl-donor supplementation in viable yellow agouti mice altered offspring DNA methylation and phenotype [28,29]; vitamin B12 deficiency altered DNA methylation in rat colonic epithelium [30]; long-term folic acid plus vitamin B12 supplementation changed genome-wide methylation in older adults [31]; dietary methionine availability has been shown to alter one-carbon metabolic state and methylation-linked chromatin regulation in experimental systems [32]; and periconceptional restriction of vitamin B12, folate and methionine altered offspring DNA methylation, insulin resistance and blood pressure in sheep [33].

One-carbon metabolism is widely recognized as having the strongest biochemical basis for linking nutrition to DNA methylation because it directly regulates methyl-donor availability and the SAM:SAH balance [14,21]. However, the level of causal support varies across study designs. Controlled animal studies provide proof-of-principle that methyl-donor availability can influence methylation and phenotype during development, while large-animal studies extend this concept to mammalian developmental programming [28,29,33]. Human supplementation studies show that methyl-donor status can modify selected methylation marks. Still, the observed effects are often modest, tissue- or cell-type-dependent, and not always linked to clear phenotypic outcomes [17,31]. Thus, one-carbon metabolism is best interpreted as an established biochemical pathway and a plausible mediator of nutrition-sensitive methylation changes, but not as uniform evidence that methyl-donor intake causally drives long-term human outcomes at all loci.

### 2.2. Metabolic State and Signaling

DNA methylation can be influenced by nutrient and energy-sensing pathways (Figure 1(2)). AMPK is a cellular energy sensor activated during energy stress and caloric restriction, and it can enhance SIRT1 activity by increasing NAD^+^ availability [34,35]. This links cellular energy status to deacetylation-dependent chromatin regulation, because SIRT1 regulates histone acetylation and can deacetylate DNMT1, altering its methyltransferase activity [36]. AMPK can also influence DNA demethylation more directly by phosphorylating and stabilizing TET2, thereby linking glucose availability to 5hmC dynamics [37]. Dietary restriction studies further support the relationship between SIRT1 activity, DNA methylation and diet-responsive gene regulation [38]. Similarly, activation of the rapamycin (mTOR) pathway elevates DNMT1 expression, promoting DNA methylation [39].

The role(s) of signaling pathways are biologically plausible, but are not as well established as one-carbon metabolism. Much of the evidence derives from experimental systems in which particular pathways have been manipulated [22,35,36,37,39]. Human dietary studies rarely integrate simultaneous measures of how the nutrient exposure impacts signaling activity and methylation changes [17]. Therefore, we contend that these mechanisms should be considered as context-dependent routes through which nutritional state may influence methylation, rather than as a single established causal pathway in humans.

### 2.3. Microbiome

Short-chain fatty acids (SCFAs), including butyrate, propionate, and acetate, are produced through gut bacterial fermentation of dietary fiber and resistant starch. Butyrate functions as an HDAC inhibitor, which alters chromatin accessibility and indirectly affects DNA methylation (Figure 1(3)) [23,24,40]. Some gut bacteria produce folate, increasing the host’s available folate levels and influencing the one-carbon metabolic pathway and DNA methylation processes (Figure 1(3)) [41].

Microbiome-mediated effects on methylation represent a promising possibility but are yet to be established causally. These effects, by definition, must occur through signaling processes. As a result, it is difficult to obtain definitive evidence for a microbial role in methylation. This is further complicated by observations that microbial composition and metabolite production vary substantially between individuals, and may be linked to diet, medication usage, geography, and health status [20]. For this reason, we contend that microbiome-linked methylation should be considered as a potential context-dependent signal of methylation status. Confirmation of these effects requires appropriately designed longitudinal intervention studies with paired microbiome, metabolite, methylation, and cell-composition data [23,24].

### 2.4. Dietary Bioactives and Micronutrients

Food-derived polyphenols (e.g., epigallocatechin-3-gallate from green tea, resveratrol from grapes and berries, and curcumin from turmeric) inhibit DNMTs in vitro, leading to DNA hypomethylation and the reactivation of silenced genes (Figure 1(4)) [42,43]. Similarly, ascorbic acid (vitamin C) is a cofactor that enhances TET enzyme activity, promoting demethylation [44]. Dietary factors that affect protein degradation pathways regulate the stability of DNMTs and TETs through post-translational modifications including phosphorylation, ubiquitination, and SUMOylation [45,46].

Micronutrients have specific effects. For example, zinc affects the structural integrity and catalytic activity of DNMTs, and thus methylation patterns [47]. Similarly, selenium and vitamin D modulate DNMT expression through antioxidant and VDR-mediated signaling, respectively, and shift methylation at redox-sensitive and immune-related loci [48]. Sulforaphane from cruciferous vegetables acts largely through HDAC inhibition, with downstream effects on chromatin accessibility that indirectly influence DNA methylation at associated promoters [49]. NAD+ availability further regulates sirtuin activity and, by extension, DNMT deacetylation and global methylation dynamics [22,50].

The translational evidence supporting the effects of dietary bioactives and micronutrients on methylation status varies. Foundational findings for the impact of food-derived bioactives often come from experimental systems (e.g., cultured cells, cancer models, enzyme assays) or exposure levels that do not reflect typical dietary intake [42,49]. Therefore, the relevance of these in vitro findings to human systems is debatable. Despite this, selected micronutrients have clear biochemical or regulatory links to methylation-related pathways [17,44,47,48].

### 2.5. Oxidative Stress, Inflammation, and Alcohol Exposure

Reactive oxygen species (ROS) directly damage DNA. Oxidative stress also decreases cellular SAM levels, thereby limiting the availability of this substrate for DNA methylation (Figure 1(5)) [51]. Dietary antioxidants, including fruits, vegetables, and plant-based bioactive compounds, reduce oxidative stress while also protecting DNA methylation integrity [16]. Suppressing inflammatory pathways through dietary intake of omega-3 fatty acids, polyphenols, and other phytochemicals helps normalize the expression and activity of DNMTs [52]. For example, inflammatory cytokine signaling can alter DNMT expression and activity in a context-dependent manner, with IL-6 linked mainly to DNMT1-mediated promoter hypermethylation and chronic inflammation-associated IL-10/STAT3 signaling linked to DNMT3B-dependent tumor-suppressor silencing [53,54]. The bacterial transformation of host-derived bile acids into secondary bile acids influences inflammatory processes and oxidative stress, as well as metabolic pathways that control DNMTs and TETs [55,56].

Alcohol consumption is an important nutritional and lifestyle exposure that should be considered separately because it interferes directly with one-carbon metabolism and methylation potential [57,58]. In experimental models, chronic ethanol exposure disrupts methionine adenosyltransferase activity, lowers hepatic S-adenosylmethionine (SAM) availability, disturbs SAM homeostasis, and can produce global or locus-specific DNA methylation changes [57,59,60]. Human epigenome-wide association studies also identify reproducible alcohol-associated DNA methylation signatures in blood, indicating that alcohol can act as an exposure, confounder, or effect modifier in nutrition-methylation analyses [61,62].

These potential effects of oxidative stress and inflammation on methylation status require careful interpretation. In human studies, methylation differences linked to inflammatory or redox states may reflect direct epigenetic regulation, but they may also capture disease activity, altered metabolism, tissue damage, or shifts in immune-cell proportions [25,51]. Moreover, while cytokine-related methylation mechanisms are biologically plausible, much of the supporting evidence comes from inflammatory disease or cancer-related models [53,54]. The evidence supporting the effects of alcohol exposure is more significant due to the existence of experimental and observational studies [57,59,61,62]. Again, we contend that inflammatory and oxidative stress pathways are potential context-dependent modifiers, and the causal links remain to be identified.

### 2.6. Response Modifiers and Emerging Routes

Genetic variants affect how individuals respond to dietary interventions and the effects of nutrition on epigenetic regulation [63]. For example, the *MTHFR* C677T polymorphism reduces MTHFR enzyme activity, limiting conversion of 5,10-methylene-THF to 5-MTHF and thereby affecting methyl-group availability for methionine and SAM-dependent DNA methylation [6,21].

Different dietary substances affect the production of microRNAs (miRNAs) that, in turn, regulate the expression of DNA methylation-related genes [64]. Evidence suggests that maternal breast milk miRNAs influence neonatal DNA methylation and gene expression patterns, although the extent of functional uptake remains under investigation [65,66]. Similarly, debate continues about whether miRNAs from food products (e.g., plants) can survive digestion to circulate throughout the body and impact epigenetic mechanisms [67,68].

## 3. How Is Epigenetics Linked to Development and Disease?

DNA methylation links development and disease through gene regulation during cell-fate specification, genomic imprinting, tissue maturation, immune and metabolic programming, and aging [5,12,69]. Developmental methylation changes help encode normal lineage commitment and tissue-specific gene regulation, but when nutritional stress occurs during sensitive windows, the same regulatory machinery may contribute to persistent changes in growth, neurodevelopment, immune function, and metabolic disease risk [70,71,72,73]. In established disease, methylation can function as an exposure marker, a marker of tissue or immune-cell composition, a mediator of altered gene expression, or a downstream consequence of inflammation and tissue remodeling [25,74,75,76,77]. Determining whether DNA methylation acts as an exposure marker, a mediator, or a downstream consequence is therefore essential for interpreting causal evidence in nutritional epigenetics.

The Developmental Origins of Health and Disease (DOHaD) hypothesis emphasizes a role for epigenetic elements, including DNA methylation, in linking early environmental exposures to adult health outcomes [71]. Emerging evidence suggests that methylation responses may vary according to sex and early-life exposures, including birth mode [78,79]. Historical cohort studies of famine survivors have demonstrated that specific methylation patterns endure for decades after the initial nutritional deprivation [72,73]. The impact of such changes, particularly if they occur during a critical developmental window (e.g., between prenatal and early postnatal life) can be enduring [80,81,82]. The enduring impacts of these changes can be further extended through intergenerational epigenetic effects, although transgenerational inheritance in humans remains uncertain [83].

## 4. How Can We Measure Nutrition-Associated Methylation Changes?

Emerging technologies enable researchers to measure native DNA methylation directly, resolve cell-type heterogeneity, and integrate methylation with complementary molecular layers (Table 1).

Long-read sequencing (e.g., Oxford Nanopore Technologies (ONT) and Pacific Biosciences (PacBio) HiFi sequencing) enables direct profiling of DNA methylation (5mC) from native DNA without bisulfite conversion [84]. This means that long-range methylation patterns across CpG islands, repetitive regions, and imprinted loci can be quantified [85,86]. In addition to detecting site-level methylation, long-read sequencing enables methylation to be phased with haplotypes or structural variants to address questions that are difficult to resolve with short-read assays [86,87]. This ability to phase directly measured methylation profiles is particularly useful in nutrition research when measuring the impacts of dietary exposures on methylation at imprinted or allele-sensitive loci.

Single-cell methylome sequencing has further expanded the study of epigenetics by revealing cell-type-specific methylation patterns that are often masked in bulk tissue data [88,89]. This matters in nutrition studies because diet, inflammation, and developmental state can all shift tissue composition, making it difficult to determine whether an observed methylation change reflects true epigenetic remodeling or a change in cell-type proportions. A major practical advance is the growing use of methylation-reference atlases and computational deconvolution frameworks to interpret bulk data, especially in blood-based studies, and to separate cell-composition effects from locus-specific methylation change [90,91].

Understanding the nutritional effects on DNA methylation is strengthened when methylome data are analyzed alongside transcriptomics, metabolomics, proteomics, and microbiome data. This allows researchers to move beyond association and ask whether methylation changes track with altered gene expression, metabolic pathway activity, or shifts in host–microbe interactions [92,93]. In practice, integrated multi-omics designs are especially useful for distinguishing exposure markers from functional mediators, for example, when a diet-associated CpG is accompanied by concordant changes in nearby gene expression, circulating metabolites, or inflammatory proteins. Machine learning and network-based approaches are increasingly used to analyze high-dimensional datasets. The expansion of single-cell methylome sequencing to co-profile epigenetics and transcriptomes in tissue sections at near single-cell resolution, creates an important future direction for nutrition research where tissue architecture may influence epigenetic response [94].

**Table 1:** Technologies for resolving nutrition-associated methylation signals.

Methods	Strength	Limitations	Application to Nutrition Studies	References
ONT long-read methylation sequencing	Native 5mC detection; haplotype phasing; repeats and structural variants	Higher error rate; high DNA input; computational cost	Imprinting; allele-specific methylation; repeat regions; longitudinal WGS	[85,86,87]
PacBio HiFi methylation profiling	High accuracy; long reads; joint variant–methylation calling	Lower throughput; high cost; fewer mature pipelines	Phase-resolved methylation; variant-linked methylation	[84]
Single-cell methylome sequencing	Cell-type resolution; developmental trajectories; rare-cell signals	Sparse CpG coverage; technical noise; limited scale	Heterogeneous tissues; development; diet-responsive cell states	[88,89,95]
Methylation atlas and deconvolution methods	Cell-composition inference; bulk-data correction; retrospective use	Reference dependence; limited rare-cell resolution	Blood studies; bulk tissues; composition adjustment	[90,91]
Spatial methylome-transcriptome profiling	Tissue context; spatial gene regulation; cell-neighbor effects	Low throughput; preservation sensitivity; early-stage methods	Gut, liver, placenta, brain; tissue-specific diet response	[94]
Multi-omics integration with machine learning	Cross-layer integration; biomarker discovery; response prediction	Sample size; overfitting; batch effects; interpretability	Mechanistic inference; intervention response; precision nutrition	[92,93,96,97]

## 5. What Limits Causal Inference in Nutritional Epigenetics?

Several limitations are impacting the ability to move from an associative to a causal understanding of the inter-relationships between epigenetics and health. Reverse causation is a critical limitation when interpreting nutrition–DNA methylation relationships. In effect, reverse causation is the problem whereby observed methylation differences are either a consequence of the physiological or disease state, or they are the causal mediators of nutritional exposure [26,77]. As noted above, features of inflammation, metabolic dysfunction, or shifts in cellular composition, each of which can be influenced by diet, can independently alter DNMT and TET activity, redox balance, and chromatin state [17,22,98]. This leads to secondary changes in the DNA methylation profile. This means that the phenotype-associated methylation signature may reflect downstream responses to altered biology rather than the direct effects of specific nutrients. This problem is particularly important in cross-sectional studies, where temporal ordering cannot be established, and in blood-based analyses, where diet-related changes in immune cell proportions can be interpreted as locus-specific methylation differences [25,75,76,99]. Distinguishing cause from consequence requires experimental designs that establish directionality, including longitudinal sampling with repeated measures, intervention studies, and analytical strategies such as Mendelian randomization and mediation analysis [26,100,101]. Without these approaches, there is a substantial risk that nutrition-associated methylation signals can be misinterpreted as mechanistic drivers when they are actually biomarkers of the physiological state.

Among the most pressing technical hurdles, conventional bisulfite-based methods for DNA methylation profiling are technically challenging because the harsh chemical treatment causes substantial DNA degradation and fragmentation and cannot distinguish 5-methylcytosine (5mC) from 5-hydroxymethylcytosine (5hmC) [102]. Similarly, long-read sequencing requires relatively large amounts of high-molecular-weight DNA and exhibits higher per-read error rates than short-read sequencing methods [103]. Single-cell methylation approaches also suffer from substantial technical noise, leading to high allelic dropout and limited genomic coverage [104]. Adding to these complexities, evaluating bulk tissue samples poses challenges because different cell types within these samples exhibit distinct methylation patterns [25]. Consequently, bulk measurements capture only an average methylation signal, which can obscure or dilute changes that occur in specific cell types.

Beyond technical limitations, the research design required to deconvolute the epigenetic impact of nutrition poses significant obstacles. Observational cross-sectional studies cannot establish cause-and-effect relationships. Compounding this issue, the primary dietary assessment typically relies on self-reported food-frequency questionnaires, which yield unreliable results due to inaccurate recall of food consumption and measurement errors [105,106].

Representation and generalizability also require careful consideration. Differences in ancestry, dietary context, environmental exposures, and study design can influence methylation estimates and limit direct comparison across populations [107]. Similarly, differences in methylation architecture, assay design, and experimental systems can affect how findings are interpreted across species or translated into human studies [108].

The analysis of multi-omic data encounters technical challenges because applying multiple tests to high-dimensional data often leads to high false discovery rates. This is further complicated by the typically small size of epigenetic datasets that have in-depth profiling. Thus, studies typically have low statistical power due to the complexity of the confounders (e.g., age, sex, genetic background, smoking, physical activity, and medication use). Yet stringent statistical corrections are not always appropriate, and the high cost of epigenomic profiling limits sampling [109,110]. Finally, even when methylation changes are detected, our ability to interpret them remains limited due to a lack of an understanding of the mechanisms for how methylation changes affect gene expression and subsequent downstream effects and during which developmental window these dietary changes were established [111].

## 6. What Does Causality Look Like When Studying Nutrition and Epigenetics?

Establishing causality in nutritional epigenetics requires distinguishing methylation as a marker of exposure from methylation as a mechanism of effect. Evidence should therefore be interpreted according to both study design and biological plausibility. In vitro studies and biochemical assays can show that nutrients, metabolites, or dietary bioactives influence DNMT activity, TET activity, chromatin accessibility, or methylation-related gene regulation, but they do not establish organism-level or clinical relevance [26,42,44].

Animal models provide stronger mechanistic support because dietary exposures can be experimentally controlled across developmental windows and tissues, although species-specific metabolism and methylation architecture limit direct translation to humans [29,33]. Observational human studies and epigenome-wide association studies can identify nutrition-associated CpGs or differentially methylated regions (DMRs), but they remain vulnerable to confounding, reverse causation, dietary measurement error, tissue specificity, and cell-composition effects [25,74,75,76].

Longitudinal cohorts and randomized dietary interventions improve temporal interpretation by showing whether methylation changes follow exposure or intervention, but they still do not prove that methylation mediates the phenotypic effect unless supported by mediation analysis, Mendelian randomization, or functional validation [26,100,101].

**Figure 2:**
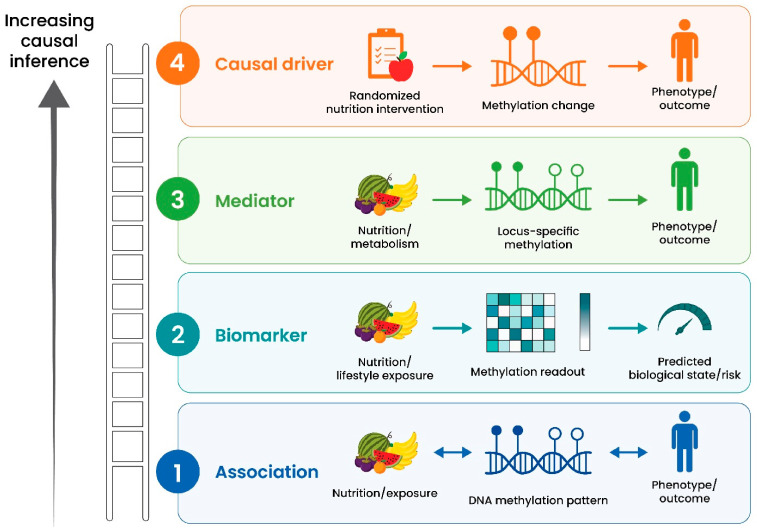
Evidence ladder for causal inference in nutrition and DNA methylation studies. Nutrition-associated methylation findings can be interpreted across four levels of evidence: association, biomarker, mediator, and causal driver. Causal inference strengthens as studies move from non-directional links between nutrition, methylation and phenotype toward randomized intervention evidence showing methylation change with phenotypic response. Filled lollipops indicate methylated CpGs; open lollipops indicate unmethylated or lower-methylated CpGs. Numbers 1–4 denote progressively stronger levels of causal evidence, and the upward arrow indicates increasing strength of causal inference.

Causality can therefore be framed as an evidence ladder, where each level asks a different question: is methylation associated with nutrition, does it mark biological response, does it lie on the pathway to phenotype, or does it drive the phenotype itself? Causal inference, therefore, strengthens as studies move from non-directional associations to methylation readouts, pathway-level mediation and randomized intervention-sensitive methylation changes (Figure 2).

At the base of the ladder, association studies show that nutritional exposures and nutritional stressors can leave detectable methylation signatures. Historical famine cohorts, Gambian season-of-conception studies, severe acute malnutrition studies, and large prenatal EWAS indicate that the methylome is sensitive to nutritional context [72,73,112,113,114,115,116,117]. These studies are valuable for discovery, but they cannot determine whether methylation is a cause, a consequence, or a correlated marker of altered development, infection, inflammation, adiposity, or cell composition (Table 2).

Randomized dietary trials strengthen inference because the exposure is assigned rather than merely observed. However, most intervention studies still support methylation primarily as a biomarker. Epigenetic clocks and differentially methylated regions have responded to caloric restriction, Mediterranean-style diets, vegan diets, fasting-mimicking diets, omega-3 supplementation, and multivitamin supplementation [118,119,120,121,122,123,124,125,126,127,128,129,130,131,132]. These findings show that nutrition can shift selected methylation readouts, but a shifted clock or a CpG alone does not, by itself, prove that methylation mediates the health benefit (Table 2).

The key distinction is between methylation as a readout and methylation as a mechanism. A dietary intervention may slow an epigenetic clock by reducing inflammation, altering immune-cell proportions, altering body weight, or improving metabolic state. These are important biological responses, but they do not establish that methylation is the causal pathway from nutrition to outcome. Mediator evidence requires stronger temporal and analytical support. Locus-specific examples in growth and metabolic biology, including *SOCS3*, *CPT1A*, *ABCG1*, *TXNIP*, and *DHCR24*, provide the clearest support for methylation, lying on a pathway between nutrition-related biology and phenotype [100,101,133,134]. Even here, the evidence is usually triangulated from EWAS, replication, Mendelian randomization, and functional plausibility rather than from nutrition RCTs that formally test mediation (Table 2).

The causal-driver tier remains the narrowest part of the evidence base. Prenatal folic-acid trials implicating *ZFP57* provide one of the strongest human examples because randomized exposure has been linked to reproducible methylation differences and later child phenotypes [135,136,137,138]. The WASH Benefits Bangladesh trial provides rare evidence from a low-income child-nutrition context, linking a combined nutrition, water, sanitation, and handwashing intervention to *NR3C1* methylation and stress physiology [139]. Nevertheless, neither example directly perturbs methylation itself. They therefore support intervention-sensitive methylation and plausible pathway involvement, but not definitive methylation-driven causality (Table 2).

Together, the current evidence supports a cautious causal framework. Nutrition is consistently associated with variation in DNA methylation, and randomized interventions show that selected methylation readouts can respond to dietary change. However, most findings remain at the level of association or biomarker response. Only a smaller set of loci shows plausible mediator evidence, and definitive causal-driver evidence remains rare. Thus, nutrition-associated methylation signatures should be interpreted in proportion to evidential strength: most are best viewed as exposure-sensitive markers or predictive biomarkers, some may represent pathway-level mediators, and only a small minority currently justify causal-driver claims.

## 7. How Can the Field Move Toward Causal Precision Nutrition?

**Figure 3:**
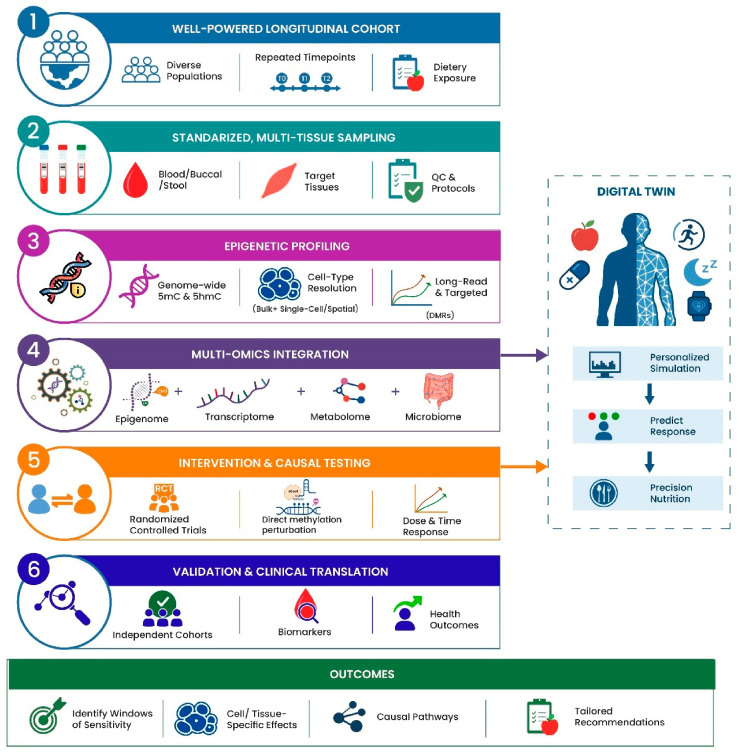
Experimental framework for future methylation-based nutrition studies. The numbered panels indicate the framework’s sequential progression. Step 1 defines a well-powered longitudinal cohort with diverse populations, repeated timepoints, and dietary exposure assessment. Step 2 emphasizes standardized multi-tissue sampling, including blood, buccal, stool, and target tissues, together with quality-control procedures. Step 3 focuses on multi-omic epigenetic profiling, including genome-wide assessment of 5mC and 5hmC, cell-type resolution across bulk, single-cell, and spatial approaches, and long-read and targeted methylation analyses. Step 4 integrates epigenomic data with transcriptomic, metabolomic, and microbiome profiles. Step 5 moves to intervention and causal testing through randomized controlled trials, direct methylation perturbation, and dose- and time-response analysis. Step 6 emphasizes validation and clinical translation through independent cohorts, biomarkers, and health outcomes. The digital twin panel represents a downstream application for personalized simulation, response prediction, and precision nutrition. The purple arrow indicates that integrated multi-omic information from Step 4 can inform personalized simulation within the digital-twin framework. The orange arrow indicates that findings from intervention and causal testing in Step 5 can inform and validate response-prediction models, which may subsequently support precision-nutrition recommendations.

The application of multimodal tools (Figure 3) to the study of epigenetics will shift the field away from associative to causal analyses, predictive modeling, and explainable artificial intelligence frameworks that prioritize biologically informative changes [96,97]. This is central to the problem of cause and effect in nutritional epigenetics, because future studies must determine whether nutrition-associated methylation changes are exposure markers, adaptive responses, mediators, or causal drivers of downstream phenotypes.

For nutrition research, long-read sequencing improves locus resolution, single-cell and deconvolution approaches improve cellular interpretation, and multi-omics integration strengthens functional inference. Application of multimodal tools to long-read data can potentially identify robust methylation signatures of diet quality, intervention response, or biological aging. An important next step for nutrition studies is to combine these tools with repeated sampling, which will determine whether methylation changes are transient, adaptive, or persistent across intervention windows, and thus enable the deconvolution of the effects of timing, tissue context, and cell composition on disease risk [99,140]. Integration of methylation data with anthropometric, inflammatory, microbiome, metabolomic, and neurodevelopmental measures should further strengthen mechanistic interpretation and biomarker discovery within precision nutrition frameworks [92,93].

Whether methylation changes can function as reliable clinical indicators of health requires cautious epidemiological interpretation and further validation across diverse populations [141]. However, future progress depends on coordinated precision nutrition study design and cross-cohort data harmonization that enable the combination of high-depth phenotyping and multimodal data that accounts for the genetic, epigenetic, environmental, and dietary variation that shapes individual intervention response [142]. Pilot diet and lifestyle intervention studies illustrate the translational potential of methylation-based outcomes, but also highlight the need to move from statistical correlation toward mechanistic validation [143]. Subsequently, the application of this knowledge to the design of randomized controlled trials for health outcomes will support the development of methylation-based biomarkers that clarify how diet shapes human development from birth to old age, define critical intervention periods, and characterize tissue- and cell-type responses [17,140,144]. This will be particularly important for malnutrition research, where the key question is not only whether methylation changes occur after nutritional stress, but whether they persist, reverse, or contribute to incomplete biological recovery. However, even longitudinal cohorts and randomized nutrition trials mainly test nutrition as the exposure; they do not directly test whether methylation itself is sufficient to alter downstream biology. The strongest causal test would require direct perturbation of methylation at the implicated locus, followed by assessment of downstream molecular or phenotypic change; this remains rare in human nutrition studies. Targeted epigenome-editing approaches, including CRISPR–dCas9 recruitment of DNMT or TET activity, provide a conceptual route to test whether methylation at a defined locus is sufficient to alter gene regulation or phenotype (Figure 3) [145,146]. Where direct perturbation is not feasible, integrated longitudinal and multi-omic datasets can help prioritize candidate methylation markers, mediators, and intervention windows for future validation.

In biology, digital twin models are data-driven representations of individuals created by integrating molecular (e.g., genomics, epigenomics), physiological (e.g., anthropometrics), clinical (e.g., disease states) and environmental data (e.g., nutrition) to simulate how the individual behaves over time (e.g., their risk for developing disease). Within this framework, digital twin models should be viewed as downstream translational applications rather than replacements for longitudinal or randomized evidence (Figure 3). Their value depends on the quality of the longitudinal, interventional and multi-omic data used to train them. In the near term, digital twin models that connect nutrition and epigenetics will simulate recovery trajectories by using in-depth longitudinal datasets. This will extend the multimodal framework for analyzing the nutrition–epigenetic relationship by enabling in silico experiments that predict individualized responses to intervention and support the targeted design of precision nutrition strategies [147,148].

## 8. Conclusions

Nutrition-associated DNA methylation changes are of major interest because some signatures persist even after recovery, with potential implications for long-term disease risk, particularly when nutritional stress occurs during sensitive developmental windows. However, persistence does not itself establish causality, and evidence for transgenerational epigenetic inheritance in humans remains uncertain. The central challenge is therefore to determine whether a methylation change reflects a non-directional association, serves as an exposure-sensitive biomarker, acts as a pathway-level mediator, or functions as a causal driver of phenotype. Progress will require longitudinal and randomized intervention studies, rigorous control of tissue and cell composition, improved methods for inferring directionality, and direct functional validation, including locus-specific perturbation where feasible. Harmonizing these methodological advances with high-resolution methylome profiling and multi-omic integration should strengthen biomarker development, clarify mechanistic pathways, and support evidence-based precision nutrition without overstating causal claims.

## Figures and Tables

**Table 2:** Evidence ladder for causal interpretation in nutrition and DNA methylation studies.

Evidence Tier	Interpretation	Sources of Evidence	Representative Examples	Main Inference Supported	Key Limitations	References
Association	Correlated with nutrition or phenotype.	Observational EWAS, cross-sectional studies, famine cohorts	Prenatal famine and *IGF2*; Gambian seasonality; one-carbon biomarkers; kwashiorkor; EMPHASIS/PACE EWAS.	Good for discovery.	Direction unclear; confounding likely.	[72,73,112,113,114,115,116,117]
Biomarker	Reflects exposure or biological state.	Randomized or repeated-measure dietary interventions; epigenetic-clock studies	DOMInO; DIRECT-PLUS; NU-AGE; CALERIE; TwiNS; FMD; DO-HEALTH; COSMOS.	Methylation responds to nutrition	Does not prove the mechanism	[118,119,120,121,122,123,124,125,126,127,128,129,130,131,132]
Mediator	May lie on the pathway to phenotype.	Mendelian randomization, mediation/pathway analysis, methylation–phenotype integration	*SOCS3* and childhood height; *CPT1A*, *ABCG1*, *TXNIP*, and *DHCR24* in metabolic traits.	Pathway-level support	Direct intervention evidence is limited	[100,101,133,134]
Causal driver	Methylation may contribute to phenotype.	Strong randomized exposure-linked loci; functional validation where available	Folic acid: *ZFP57*; WASH Benefits: *NR3C1*.	Strongest causal support	Rare in human nutrition	[135,136,137,138,139]

## Data Availability

No new data were created or analyzed in this study. Data sharing is not applicable to this article.
